# Decreased IL-6 and NK Cells in Early-Stage Lung Adenocarcinoma Presenting as Ground-Glass Opacity

**DOI:** 10.3389/fonc.2021.705888

**Published:** 2021-09-08

**Authors:** Pengfei Zhang, Boxue He, Qidong Cai, Guangxu Tu, Xiong Peng, Zhenyu Zhao, Weilin Peng, Fenglei Yu, Min Wang, Yongguang Tao, Xiang Wang

**Affiliations:** ^1^Department of Thoracic Surgery, Second Xiangya Hospital, Central South University, Changsha, China; ^2^Hunan Key Laboratory of Early Diagnosis and Precision Therapy, Second Xiangya Hospital, Central South University, Changsha, China; ^3^Department of Laboratory Medicine, Second Xiangya Hospital, Central South University, Changsha, China; ^4^Key Laboratory of Carcinogenesis and Cancer Invasion, Ministry of Education, Department of Pathology, Xiangya Hospital, Central South University, Changsha, China; ^5^National Health Commission of the People's Republic of China (NHC), Key Laboratory of Carcinogenesis (Central South University), Cancer Research Institute and School of Basic Medicine, Central South University, Changsha, China

**Keywords:** early lung adenocarcinoma, IL-6, NK cells, tumor microenvironment, ground-glass opacities

## Abstract

**Background:**

Lung ground-glass opacities (GGOs) are an early manifestation of lung adenocarcinoma. It is of great value to study the changes in the immune microenvironment of GGO to elucidate the occurrence and evolution of early lung adenocarcinoma. Although the changes of IL-6 and NK cells in lung adenocarcinoma have caught global attention, we have little appreciation for how IL-6 and NK cells in the lung GGO affect the progression of early lung adenocarcinoma.

**Methods:**

We analyzed the RNA sequencing data of surgical specimens from 21 patients with GGO-featured primary lung adenocarcinoma and verified the changes in the expression of IL-6 and other important immune molecules in the TCGA and GEO databases. Next, we used flow cytometry to detect the protein expression levels of important Th1/Th2 cytokines in GGO and normal lung tissues and the changes in the composition ratio of tumor infiltrating lymphocytes (TILs). Then, we analyzed the effect of IL-6 on NK cells through organoid culture and immunofluorescence. Finally, we explored the changes of related molecules and pathway might be involved.

**Results:**

IL-6 may play an important role in the tumor microenvironment of early lung adenocarcinoma. Further research confirmed that the decrease of IL-6 in GGO tissue is consistent with the changes in NK cells, and there seems to be a correlation between these two phenomena.

**Conclusion:**

The IL-6 expression status and NK cell levels of early lung adenocarcinoma as GGO are significantly reduced, and the stimulation of IL-6 can up-regulate or activate NK cells in GGO, providing new insights into the diagnosis and pathogenesis of early lung cancer.

## Introduction

Lung cancer is the leading cause of cancer-related mortality worldwide, killing an estimated 1.6 million people each year, with 5-year overall survival rates ranging from 85% in stage IA to 6% in stage IV (1). Thanks to high-resolution computed tomography (HRCT), the diagnosis of ground-glass opacity or ground-glass nodules (GGO or GGN) found in the surrounding lung field is increasing (2, 3). GGO is defined radiologically as a focal lesion that visually preserves lung parenchyma, airways, and blood vessels in it (4). GGO can be regarded as the symbol of both benign or malignant lesions, and its occurrence and growth are involved in various molecular changes (5). Many of these GGOs are associated with early lung adenocarcinoma (LUAD) which is the dominant histologic subtype in lung cancer (2). A consensus on the importance of preoperative characterization of lesions has been reached. The molecular and cellular changes in GGO may provide new insights into the pathogenesis of early lung adenocarcinoma.

Abnormality in the immune microenvironment is one of the most important characteristics of cancer initiation and progression. For example, the expression of the Programmed Death-Ligand 1 (PD-L1) stimulated by the PD-1/PD-L1 axis is a major immunosuppressive mechanism in non-small cell lung cancer (NSCLC) (6, 7). Some tumor-infiltrating lymphocytes (TILs) also are closely tied with cytokines in the tumor microenvironment (8). Many preclinical and clinical studies have shown that tumor-infiltrating lymphocytes (TILs) such as CD3+ cells, CD8+ cells, or CD45RO+ cells have the potential to be used as prognostic markers (9). Besides, in addition to immune cells, cytokines such as interleukins (IL)-4 and IL-6 also play a prominent role in the process of tumor immunity (8, 10). According to the theory of the cancer-immunity cycle, multistep processes along with various molecules and immune cells are involved in lung cancer such as the release of inflammatory cytokines, the recruitment of immune cells, the recognition of immune cells with tumor cells, and so on (11, 12). It will be more accurate and reliable to consider more steps in this cycle, that is, find more valuable biomarkers and further analyze their impact on pathological types and disease prognosis.

## Materials and Methods

### Acquisition of Clinical Samples

All specimens were collected from the specimen bank of our center (Department of Thoracic Surgery, Second Xiangya Hospital, Central South University) by members of this project.

The specimens collected met the following conditions: (1) The GGO tissue and paired normal lung tissue were surgically resected and got reserved in liquid nitrogen or got intervention immediately. The GGO tissue was collected from the middle of the primary lesion, and each pair of normal lung tissue and lung cancer tissue were from the same patient; (2) The diameter of each sample was more than 5 mm and less than 2 cm. The volume of each sample was less than 1/2 of the volume of the primary lesion; (3) All the collected specimens were confirmed as lung adenocarcinoma (LUAD) by the Department of Pathology; (4) The patients had not received any form of radiotherapy, chemotherapy, targeted drug therapy, immune drug therapy, and other tumor therapies before the operation; (5) The patients had no special systemic diseases or other diseases that affected the experimental results; (6) Specimens were obtained with the informed consent of the patients and the approval of the ethics committee.

At the same time, we collected some clinical characteristics of the GGO patients in the cohort, including age, gender, smoking history, differentiation, TNM stage, the expression of PD-L1.

### Study Cohort and RNA-seq

Totally, 73 primary lung GGO patients intended for surgical removal at the Thoracic Surgery Department of the Second Xiangya Hospital were involved in our study between February 2020 and August 2021. Because that the GGO size limited repeated usage of a lot of specimens, we finally utilized 21 pairs of tissues for RNA-seq, 26 for flow cytometry (in which a representational one for immunohistochemical staining), 1 for organoid culture, and 25 for qPCR. All the GGO samples were demonstrated to be early lung adenocarcinoma (mostly IA stage) pathologically. A panel of RNA sequencing (RNA-seq) was performed by BGI Gene Biological Company (Wuhan, China, http://www.genomics.cn/) with 21 pairs of surgical GGO specimens and normal lung tissues. All patients allowed specimen collection, clinical data provision, and biomarker analysis by written informed consent prior to enrolling in the study. We completed the study according to the Declaration of Helsinki with a protocol approved by the Ethics Committee of the Second Xiangya Hospital, Central South University, Changsha (Project identification code: 2020S609).

### Flow Cytometry

Flow cytometry was performed as per manufacturer’s protocol asked. BD Multitest CD3/CD8/CD45/CD4 reagent and BD Multitest CD3/CD16+CD56/CD45/CD19 reagent (BD Bioscience, CA, USA) were used to measure the lymphocyte percentage. Human Th1/Th2/Th17 Phenotyping Kit (Cell-Genebio, China) was used for the determination of IL-2, IL-4, IL-6, IL-10, TNF-α, IFN-γ, and IL-17a. All tests were performed on FACSCalibur (BD Biosciences, USA) instruments. The software FlowJo (LLC, version 10.6.0) was used for the data analysis.

### Data From TCGA, GEO, and Other Literature

To verify the flow cytometry results of IL-6 in lung adenocarcinoma, common shared RNA-seq data of LUAD were selected from the TCGA (The Cancer Genome Atlas) database (https://portal.gdc.cancer.gov/). The data were gotten from 59 normal cases and 535 tumors. GSE40419 RNA-seq dataset was selected from GEO (Gene Expression Omnibus) database, among which 77 normal samples and 87 LUAD tumor samples were selected, and FPKM (Fragments Per Kilobase per Million) was used to demonstrate the expression. To reinforce the generality of the results in GGO, we cited shared data in a study of lung GGO from Lee H et al. (13). In this study, researchers did a RNA-seq between 9 pairs of normal lung tissue and GGO tissue, and the pathological results of all the 9 GGO tissues are lung adenocarcinoma.

### Multiple Staining Immunohistochemical

Immunohistochemical staining was performed on 10% formalin-fixed and paraffin-embedded tissues. Antibodies of CD16 (Anti-CD16: Abcam, ab246222, at a dilution of 1:100), CD56 (Anti-NCAM1: Abcam, ab75813, at a dilution of 1:100), IL-6 (Anti-IL6: Bioworld, MB9296, at a dilution of 1:50), and PD-1 (Anti-PD-1: Abcam, ab137132, at a dilution of 1:250) were used. By the way, in the stained sections PD-1 was negative, we predicted that there is not any PD-1 expression in the selected samples, so it was not reflected in the text. The mean gray value method was used for the quantification of immunohistochemistry and immunofluorescence by ImageJ (NIH 64-bit Java 1.8.0).

### Organoid Culture and Immunofluorescence

After surgical removal of fresh GGO tissue and normal lung tissue, each tissue block is clipped into about 200mg. The specific steps of organoid culture were shown in **Figure S1**, where IL-6 was applied by PEPROTECH (Recombinant Human IL-6, Catalog:200-06, USA, at the concentration of 2ng/ml). The culture scaffolds (Millipore, PIHP01250, USA) were used as containers. Small molecules added in culture: EGF, FGF-10, FGF-basic and HGF (PEPROTECH, USA), N2, B27 (ThemerFisher, USA). Tissues were collected for immunofluorescence followed by the standard protocol. Two antibodies CD16 (Proteintech, 16559-1-AP, China, at the dilution of 1:100), and CD56 (Proteintech, 14255-1-AP, China, at the dilution of 1:3000) were used. The administration of IL-6 and controlled PBS for three groups is with 0h/24h/48h duration.

### RNA Isolation and Quantitative Real‐Time PCR

Total cellular RNA was isolated from tissue samples by Trizol reagent (Invitrogen) and reversely transcribed into cDNA using the SuperScript First Strand cDNA system (Invitrogen) according to the manufacturer’s protocol. The qPCR amplifications were performed in an Applied Biosystems Stepone Plus System (Applied Biosystems, Foster, CA) using an SYBR Green PCR Master Mix (Roche, Indianapolis, IN). Primer sequences used for performing qRT-PCR are as follows:

IL-6 forward, 5′-CACTGGTCTTTTGGAGTTTGAG-3′;

IL-6 reverse, 5′-GGACTTTTGTACTCATCTGCAC-3′;

CD16 forward, 5′-GGTGACTTGTCCACTCCAGTGT-3′;

CD16 reverse, 5′-ACCATTGAGGCTCCAGGAACAC-3′;

CD56 forward, 5′-CATCACCTGGAGGACTTCTACC-3′;

CD56 reverse, 5′-CAGTGTACTGGATGCTCTTCAGG-3′;

PD-1 forward, 5′-AAGGCGCAGATCAAAGAGAGCC-3′;

PD-1 reverse, 5′-CAACCACCAGGGTTTGGAACTG-3′;

PD-L1 forward, 5′-CGTTGTGCTTGAACCCTTGA-3′;

PD-L1 reverse, 5′-ACACAAGGAGCTCTGTTGGA-3′;

JAK1 forward, 5′-GAGACAGGTCTCCCACAAACAC-3′;

JAK1 reverse, 5′-GTGGTAAGGACATCGCTTTTCCG-3′;

STAT3 forward, 5′-CTTTGAGACCGAGGTGTATCACC-3′;

STAT3 reverse, 5′-GGTCAGCATGTTGTACCACAGG-3′;

β-actin forward, 5′-AAAGACCTGTACGCCAACAC-3′;

β-actin reverse, 5′-GTCATACTCCTGCTTGCTGAT-3′.

Results are expressed as mean ± SD of three independent experiments.

### Statistical Analysis

All data were analyzed by SPSS 22.0 software (SPSS, Chicago, IL) and plotted by GraphPad Prism 8.0 software (GraphPad Software, La Jolla, CA). Paired T-test was performed to obtain p values between two groups when complete one-to-one correspondence, otherwise the unpaired t-test was used. A p > 0.05 was considered statistically nonsignificant (ns), while the statistical difference levels were set at *P < 0.05; **P < 0.01; ***P < 0.001; ****P < 0.0001.

## Results

### Genes in Immune Regulation Behaved Abnormally in Lung GGO Tissues

**Figure 1:**
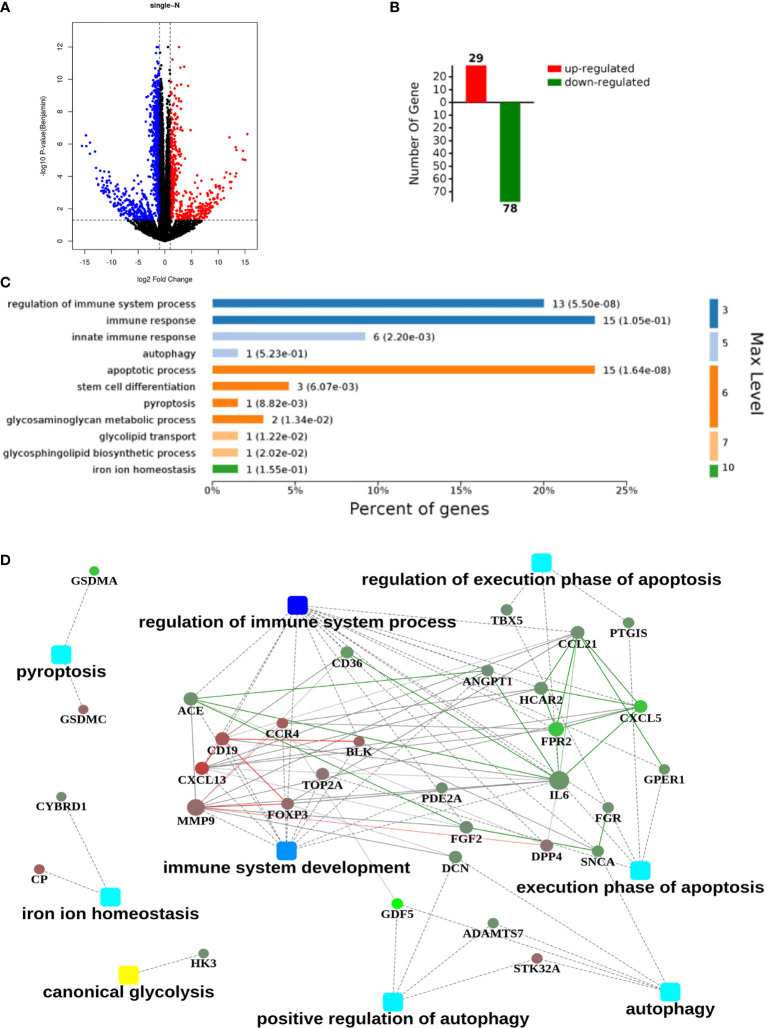
An RNA sequencing (RNA-seq) analysis with 21 pairs of surgical GGO specimens and normal lung tissues from primary lung GGO patients. **(A, B)**. Process of obtaining the up-regulated and down-regulated genes after RNA-seq in GGO (|Fold Change|≥2, p<0.05). **(C, D)**. Clustering of differentially expressed genes and the related hub genes in different pathways. Square nodes represent different pathways and round nodes represent hub genes; the size of the circle represents the number of nodes involved in hub genes.

After screening the results of RNA-seq, 1654 differentially expressed genes between normal and GGO tissues were obtained (|FC|≥2, p < 0.05), of which 572 were up-regulated in GGO and other 1082 were down-regulated (**Figure 1A, B**). It was illustrated that most of the down-regulated genes involved in immune regulation varied greatly, whereas the interleukin 6 (IL-6), CXCL13, MMP9, and some other genes seem to take crucial roles in related gene pathway models (**Figure 1C, D**). However, previous studies have shown that blocked IL-6 can inhibit the progression of some lung cancers (14), which is obviously inconsistent with our conclusion. Thus, we tried to take further study to explore this contradiction.

### The Lower Expression of IL-6 In Lung GGO Tissues

**Figure 2:**
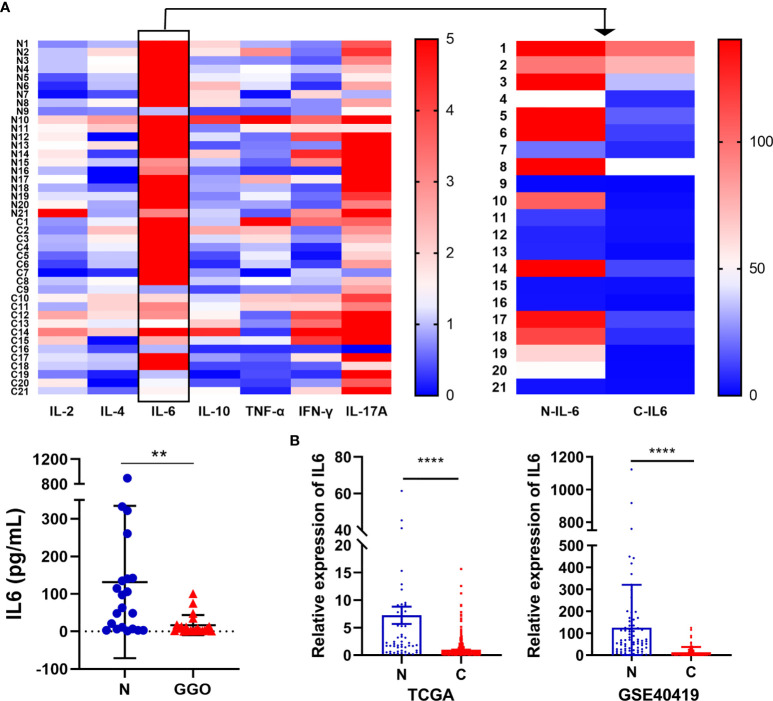
The decrease of IL-6 in lung adenocarcinoma with GGO. **(A)** The flow cytometry result of several cytokines. The heatmap shows the expression status of 7 cytokines in 21 GGO patients (upper), while the scatter plot shows the expression of IL-6 (lower). **(B)** The mRNA level of IL-6 in TCGA (left, 59 normal and 535 cancer) and GEO (GSE40419) database (right, 77 normal and 87 cancer). Each dot represents an individual patient. Results are expressed as mean ± SEM. **P < 0.01; ****P < 0.0001. N, normal; C, cancer..

We selected 21 pairs of fresh normal lung tissue and lung GGO tissue samples, extracted their proteins *in vitro*, and detected the content of IL-2, IL-4, IL-6, IL-10, TNF-α, IFN-γ, and IL-17a by cytometric bead array (CBA) microsphere method of flow cytometry (**Figure 2A** and **Supplementary Table S1**). The results showed that the expression of IL-6 in normal tissues was significantly higher than that in GGO tissues (P = 1.2e-2). Then we tried to discuss whether several important clinical characteristics affect the decrease of IL-6 in GGO patients. The result shows that age (P=0.897), gender (P=0.609), smoking history (P=0.993), differentiation (P=0.476), T stage (P=0.691) and PD-L1 (P=428) do not influence the downregulation of IL-6 (**Table 1**). To explore whether the changing trend of IL-6 level in lung adenocarcinoma from public databases is the same as our experimental result, we queried the mRNA expression of IL-6 in The Cancer Genome Atlas (TCGA) database and Gene Expression Omnibus (GEO, GSE40419)dataset—whose samples are also tissues rather than serum—and found a consistent result that the IL-6 is down-expressed in lung adenocarcinoma, where P value equals to 7.21e-26 and 4.29e-07, respectively (**Figure 2B** and **Supplementary Table S2**). Then we explored the shared transcriptome sequencing results from Lee H et al. In this cohort which included 9 normal tissues and 9 GGO tissues, the expression of IL-6 seems decreased in GGO as we predicted (**Supplementary Figure S2**).

**Table 1:** The fold change (FC) of IL-6 expression level with main characteristic of the patients in the flow cytometry.

Characteristics	n	Average FC of IL-6	P value
Age			0.897
<60	11	0.299	
≥60	10	0.230	
Gender			0.609
Female	15	0.299	
Male	6	0.230	
Smoking history			0.993
No	17	0.279	
Yes	4	0.280	
Differentiation			0.476
Well	8	0.224	
Else	13	0.313	
T stage			0.691
T1a	8	0.436	
T1b	13	0.517	
PD-L1			0.428
Negative	6	0.207	
Positive	12	0.307	

### The Amount of NK Cells Is Down-Regulated in Lung GGO Tissues

**Figure 3:**
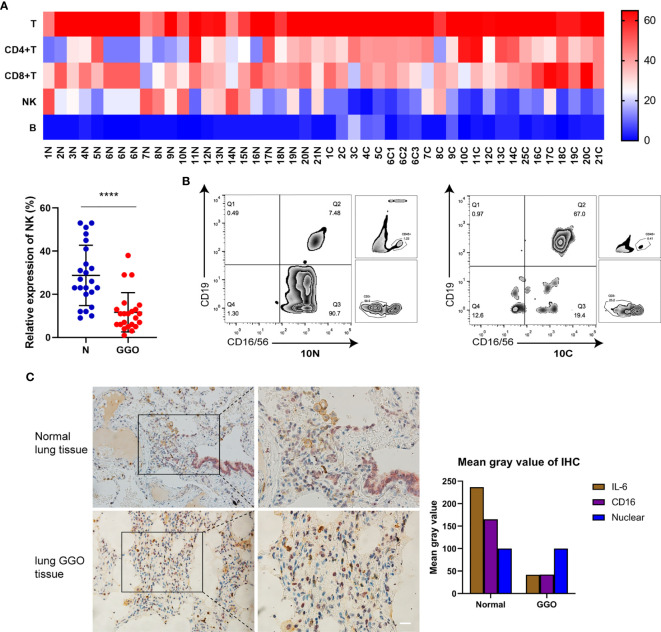
The decrease of NK cells in lung adenocarcinoma with GGO. **(A)** The flow cytometry result of immune cells. The heatmap shows the expression status of 5 kinds of cells in 21 GGO patients (upper), and the scatter plot shows the expression of NK cells (lower). **(B)** The expression of NK cells (CD16/CD56+) on total CD45+CD3- cells within the lymphocyte gate from one representative patient with paired normal (left, 10N) and GGO (right, 10C) tissues. In the paired samples selected, NK cells in normal lung tissues made up about 90.7% of all non-T lymphocytes, while the content in GGO tissues was 19.4% around. **(C)** The multiple staining immunohistochemical results of IL-6 (in brown) and CD16 (in purple) in normal lung tissue or GGO lung tissue. Each dot represents an individual patient. Results are expressed as mean ± SEM. ****P<0.0001. N, normal; C, cancer. Scale bar, 50 µm.

To simultaneously explore the changes of immune cells in the GGO tumor microenvironment, we got single-cell suspensions from 23 GGO tissues and the paired normal tissues, then applied cell differentiation by flow cytometry (**Figure 3A, B** and **Supplementary Table S3**). The 6^th^ patient had 3 GGO tissues, and 16 of the other patients got overlapped in the cytokines test. We examined the percentages of T cell (CD45^+^CD3^+^), CD4^+^ T cell (CD45^+^CD3^+^CD4^+^), CD8^+^ T cell (CD45^+^CD3^+^CD8^+^), B cell (CD45^+^CD3^-^CD19^+^ CD16/CD56^-^), and natural killer (NK) cell (CD45^+^CD3^-^CD19^-^CD16/CD56^+^) in the total cell count, where the proportion of NK cells in normal lung tissues was statistically higher than that of GGO tissues (P = 9.2e-6), whereas the proportion of T cells (P = 7.4e-4), CD4 cells (P = 2.3e-4), and B cells (P = 6.5e-3) were all increased in GGO tissues. We also explore whether some clinical characteristics could affect the decrease of NK cells in GGO patients. The result shows that age (P=0.347), gender (P=0.263), smoking history (P=0.689), differentiation (P=0.528), T stage (P=0.636) and PD-L1 (P=267) do not influence the change of NK cells in GGO tissues (**Table 2**).The multiple staining immunohistochemical experiment and its quantified results also verified the differential expressions of IL-6, CD16, and CD56 between normal and GGO tissues (**Figure 3C** and **Supplementary Figure S3**), These results further indicated the decreases in IL-6 and some important NK cell markers at the protein level in GGO.

**Table 2:** The fold change (FC) of NK cell expression level with main characteristic of the GGOs in the flow cytometry.

Characteristics	n	Average FC of NK cells	P value
Age			0.347
<60	12	0.391	
≥60	11	0.581	
Gender			0.263
Female	13	0.580	
Male	10	0.354	
Smoking history			0.689
No	18	0.503	
Yes	5	0.405	
Differentiation			0.528
Well	9	0.402	
Else	14	0.533	
T stage			0.636
T1a	10	0.416	
T1b	13	0.517	
PD-L1			0.267
Negative	8	0.333	
Positive	12	0.588	

Patient No.6 had 3 GGO nodules, which were included and calculated three times.

### The Effect of IL-6 on NK Cells

**Figure 4:**
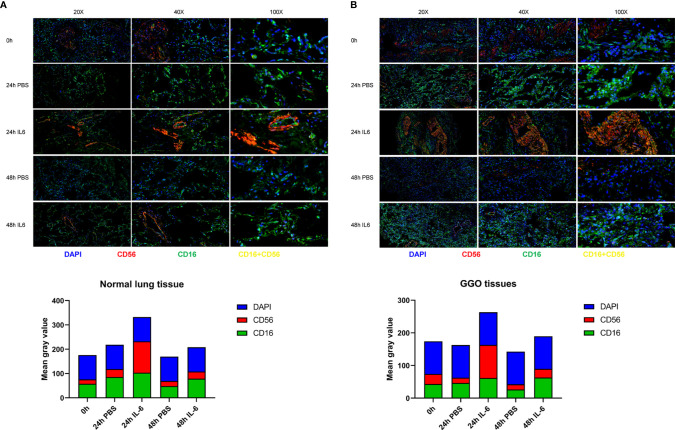
The correlation between the expression of IL-6 and NK cells in lung adenocarcinoma with GGO. Images (upper) and the mean gray value (lower) show the immunofluorescence results in GGO tissues **(A)** and their paired normal tissues **(B)** after IL-6/PBS treatment.

**Figure 5:**
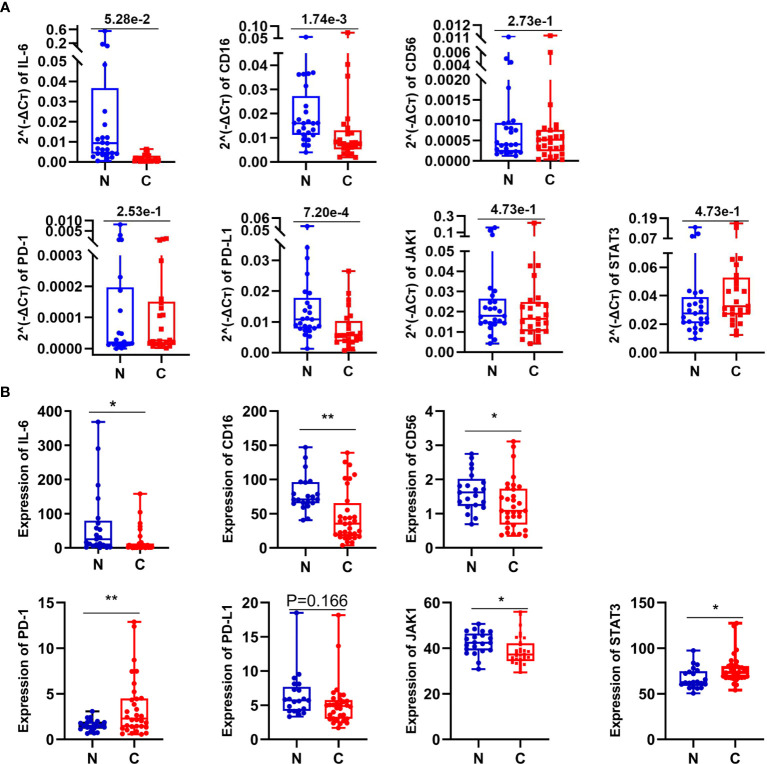
The expression of some related molecules in lung GGO tissues. **(A)** Relative expression of seven genes in 25 paired normal and cancer tissues by qPCR. **(B)** The expression of seven genes reflected by RNA-seq data come from 21 GGO patients. Each dot represents an individual patient. Results are expressed as mean ± SEM. *P < 0.05; **P < 0.01. N, normal; C, cancer.

It has been preliminary elucidated that IL-6 secreted by tumor cells could restrict the activity and function of NK cells through the JAK1 pathway (15). However, the inconsistent changes in PD-1 and STAT3 are intriguing and seem to be incompatible with the classical IL-6 pathway, which requires further research. The dysfunction of NK cells favors tumor immune-evasion, so a comprehensive understanding and restoration of their functions mechanically will aid the treatment of lung cancer (16). Consequently, we analyzed the effect of IL-6 on NK cells preliminary by organoid tissue culture with normal lung tissue and lung GGO tissue from the same characteristic patient *in vitro*. Compared to the control group (treated with PBS), the markers of NK cells—CD16 and CD56—were up-regulated in the IL-6-treated group (**Figures 5A, B**). By quantifying the results of immunofluorescence (calculated by the mean gray values of different fluorescence channels), we found that there were consistent changes in normal lung organoid tissue, but the effect of IL-6 in increasing NK cells was more obvious in GGO organoid tissue (**Figures 4A, B**). Besides, considering the importance of NK cells for checkpoint immunotherapy (17), we analyzed gene expression data and overall survival information from TCGA, finding that the lower expression of NCAM1 (CD56), NKG2D, and NCR3 (three important markers of NK cells) indicated a poor prognosis of the lung adenocarcinoma (P < 0.05, **Figure S4**). This laterally reflects the effect of decreased NK cells on tumor progression.

### IL-6/JAK/STAT3 Pathways Function in Lung GGO Tissues

To further reveal whether these involved mechanisms mentioned before also are related to IL-6 and NK cells in early lung adenocarcinoma with GGO or not, we then compared the expression of seven genes (IL-6, CD16, CD56, PD-1, PD-L1, JAK1, and STAT3) with 25 pairs of early lung adenocarcinoma samples by quantitative real-time PCR (qPCR) (**Figure 5A** and **Supplementary Table S4**) and analyzed their expression data in RNA-seq (**Figure 5B** and **Supplementary Table S5**). Even though the relative expression of CD56 by qPCR did not have significant difference (P=2.73e-1 in qPCR, and P=1.66e-1 in RNA-seq), the expression of CD16 (P=1.74e-3 in qPCR, and P=1.42e-3 in RNA-seq) and IL-6 (P=1.37e-02 in RNA-seq, although P=5.28e-2 in qPCR) showed the consistent results by qPCR and RNA-seq both. We also found that PD-L1 (P=7.20e-4 in qPCR, although P=1.66e-1 in RNA-seq) and JAK1 (P=4.74e-2 in RNA-seq, although P=4.73e-1 in qPCR) seems to be down-regulated in GGO, while PD-1 (P=8.70e-3 in RNA-seq, although P=2.53e-1 in qPCR) and STAT3 (P=1.76e-2 in RNA-seq, although P=4.73e-1 in qPCR) seems to be over-regulated in GGO.

## Discussion

Through the whole research process, we mainly drew two main conclusions: First, in the microenvironment of lung adenocarcinoma tissue with early lesions of GGO, IL-6 and NK cells showed a consistent decrease; Second, the decrease of NK cells is correlated with IL-6, and the proliferation or activation of NK cells can be stimulated by increasing IL-6 in the GGO tumor microenvironment. However, the specific mechanism of the increase of NK cells caused by IL-6 still needs to be further studied.

Interleukin-6 (IL-6) has been shown its significant characteristics in the pathological processes of inflammation, autoimmunity, and a series of cancers since its identification in the 1980s (18, 19). As a typical cytokine member of the IL-6 family, it was originally taken as B-cell stimulating factor-2 (BSF-2) which functions in immunoglobulin production (20). IL-6 binds to responding cells and takes various biological roles on them by working with transmembrane IL-6 receptors, as well as some soluble IL-6 receptors. It was widely accepted that IL-6 mainly exerted its effect through activating Janus kinase (JAK) family tyrosine kinases with the help of gp130, further causing the activation of regulatory factors such as signal transducer and activator of transcription (STAT) family transcription factors (mainly STAT3) and Src homology region 2 domain-containing phosphatase 2 (SHP-2) (21, 22). In previous studies, IL6 was generally considered as an important cancer-promoting molecule. To be more specific, IL-6 has been demonstrated to be responsible for VEGF-dependent angiogenesis in cervical cancer (23); IL-6–activated STAT3 helps cell survival and promotes cell proliferation during colitis-induced tumorigenesis of gastrointestinal cancer (24); IL-6-induced epithelial-mesenchymal transition (EMT) is critical for the metastasis of breast cancer (25) and head and neck cancers (26). As for lung cancer, IL-6 was thought to promote cancer malignancy with the functions in migratory, chemotactic, and angiogenetic properties of cancer cells by USP24 (27); microRNA-218, a down-regulated miRNA that targets the IL-6/STAT3 pathway, was illustrated to suppress lung cancer (28); meanwhile, IL-6 blockade could reduce tumorigenesis in a kind of LUAD mouse model (29). A prospective study conducted by Barrera et al. demonstrated that there are higher levels of IL-6 in the plasma of patients with non-small-cell lung cancer (NSCLC) comparing to controls (p = 0.001) (30), while a retrospective analysis by Ryan et al. also showed IL-6 has a significant association with worse survival (hazard ratio, 1.33; 95% confidence interval, 1.08-1.64; p = 0.007) with hundreds of European American lung cancer patients (31). The information above all emphasized the positive relationship between IL-6 expression and cancer development. However, as we confirmed repeatedly, IL-6 was decreased in lung adenocarcinoma with GGO. So, there seems to be a contradiction in the early tumor microenvironment regarding the change of IL-6 level. We speculate that there is a different immune response in early lung GGO tissues than in solid tumors. It was reported that in the early stages of tumor growth, immune suppression decreases immune surveillance (32). It has also been shown that some tumor suppressor cells appear in the tumor microenvironment in the early stage of tumor development (33). Obeid E et al. found that a kind of important TILs—TAMs—initially infiltrated at the tumorigenesis site as a tumor-inhibitory phenotype (M1), and then transformed into tumor-promoting macrophages (M2) in the tumor microenvironment (34). These results indicated that the content of some immune components differed in different stages of the tumor.

Natural killer (NK) cells belong to the innate lymphoid cell (ILC) family that was initially described in 1973 (35, 36). They are of lymphoid origin and recognize MHC Class I (MHC-I) molecules by their cell surface inhibitory receptors (37). According to the expression of two markers (CD56 and CD16), NK cells are classified into two main subsets: CD56^dim^ CD16^+^ NK cells and CD56^bright^ CD16^−^ NK cells. The former subset is a mature cytotoxic group that accounts for the majority of circulating NK cells, while the latter subset is less mature, less cytotoxic, primarily immunomodulatory, and mostly be found in secondary lymphoid organs (38). It was well demonstrated that NK cells function in tumor immunosurveillance as they can kill cancer cells without prior sensitization (39–41). As a kind of important tumor-infiltrating lymphocytes (TILs), low-expressed NK cells are discovered on multiple cancers, which are linked with a poor prognosis of patients, too (42, 43). A single cell sequencing study of lung GGO and solid nodules concluded that NK cell cytotoxicity was lower in solid nodules, which showed that the function of the NK cell decreased during the progression of lung adenocarcinoma (44). Mechanism researches also indicate the functional improvement of NK cells may induce tumor regression (36, 45). A study on NSCLC showed there are fewer NK cells in cancer tissues than in normal lung tissues followed by obviously reduced cytolytic potential, which mostly be caused by the decrease of CD56^dim^ CD16^+^ NK cells (46). Another study also reported that NK cells principally infiltrate the tumor stroma of lung cancer, and low levels of NK cells are associated with a bigger primary cancer size, history of tobacco smoking, and poorer prognosis (47). Specifically, we noticed that natural killer (NK) cells have the potential to be key lymphocytes involved in the early stage of lung adenocarcinoma with GGO. The expression level of CD16 and CD56 tested by qPCR and RNA-seq also suggested that the number of NK cells mostly changed in the CD56^dim^ CD16^+^ subset. By the way, the decrease of NK cells could indicate a poorer overall survival status of LUAD patients, which further highlights its clinical significance.

By searching the literature, we found that IL-6 can affect the binding of PD-L1 and PD-1 on immune cells through the IL-6/JAK1/STAT3 pathway and mediate the immune escape of tumor cells, where IL-6 up-regulates PD-L1 by the glycosylation of PD-L1 (48). And in castration-resistant prostate cancer cells (CRPC), IL-6 knockdown would lead to the low expression of related proteins in the JAK/STAT3 pathway, which could down-regulate PD-L1 in CRPC cells and reduce its binding to PD-1 on the surface of NK cells, thus affecting the content of NK cells in the tumor microenvironment (49). It was also reported that JAK1 induces glycosylation of PD-L1 by phosphorylating PD-L1 protein-related sites (Y112), thereby promoting the stability of PD-L1, while IL-6 up-regulates PD-L1 by regulating the glycosylation of PD-L1. Therefore, IL-6 can affect the stability of PD-L1 and its binding to PD-1 on immune cells through the IL-6/JAK1/STAT3 pathway, thereby mediating the immune escape of tumor cells (48).

Through organoid culture, we concluded that exogenous addition of IL-6 to lung GGO tissues could stimulate the high expression of markers (CD16 and CD56) on the surface of NK cells. However, we have not determined whether IL-6 affects the content of NK cells or just activates the cytotoxic NK cells. IL-6 has been proved to directly improve the proliferation, cytotoxicity, and other important functions of NK cells (50). Research has shown that IL-6, which is abundant in the serum of patients got some infectious diseases, may downregulate NKG2D on NK cells, leading to impaired NK activity (51–53). However, it has also been demonstrated that down-regulation of IL-6 may also block IL-6-mediated NK cell activation through the effect of IL-2 or KIR2DL1 (54–56). The combined action of cytokine IL-6 and PGL-2 can reduce the immune factor IL-2 produced by Th1 cells and affect the activation of NK cells (57). These studies indicate that IL-6 may affect the content and function of NK cells through a variety of pathways, but its role in GGO still needs further experimental verification.

In this study, we briefly explored a possibly related pathway as well as several molecules we were interested in. Results illustrated that STAT3 may be influenced during the tumorigenesis process of lung GGO as it is expressed higher in the tumor tissues than in normal lung tissues, while the trend of JAK1 seems to be consistent with that of IL-6. Therefore, the hyperactivation of STAT3 maybe not according to the well-known IL-6/JAK/STAT3 pathway (45). The STAT3 activation and signaling work through a variety of mechanisms not only related to IL-6, as SRC and some autocrine stimulation of growth factor receptors like EGFR can also lead to its induction (45). Besides, increased PD-1 and decreased PD-L1 seem to occur in the GGO tissues of lung adenocarcinoma, suggesting a probable molecular link with other changes in the immune microenvironment. The effect of IL-6 on NK cells also illustrated that there should be some underlying signaling pathway about IL-6 and NK cells in GGOs.

In summary, our study finds a significant decrease in IL-6 expression of early lung adenocarcinoma with GGO, along with the level of NK cells. It is a novel perspective that the stimulation of IL-6 can up-regulate NK cells in GGO. However, there are still some important points in the study that need to be further explored experimentally. First, why does IL-6 decrease occur in early lung adenocarcinoma manifested as GGO? We assume that it may be related to the overall low inflammatory response changes in the exceedingly early tumor microenvironment. Second, whether IL-6 affects NK cells by changing the number of NK cells in TME or by modulating the function of their surface receptors? Which may be verified by regulating the function of surface receptors.

The roles of IL-6 and NK cells in the production and development of GGO provide new insights into the early diagnosis and pathogenesis of lung adenocarcinoma. We believe further research could provide potential diagnostic biomarkers or available therapy targets for lung GGO and even take advanced benefits for studies about specific immune mechanisms in early lung adenocarcinoma.

## Data Availability Statement

The original contributions presented in the study are publicly available. This data can be found here: https://github.com/xxeyywx/RNA-seq-GGO.git. Publicly available datasets were analyzed in this study. This data can be found in **Supplementary Material**.

## Ethics Statement

The studies involving human participants were reviewed and approved by the Ethics Committee of the Second Xiangya Hospital, Central South University, Changsha. The patients/participants provided their written informed consent to participate in this study. Written informed consent was obtained from the individual(s) for the publication of any potentially identifiable images or data included in this article.

## Author Contributions

All authors contributed to the article and approved the submitted version. YT and XW conceived and designed the work. MW helped to conduct the flow cytometry. Material preparation, experiment, data collection, and analysis were performed by PZ and BH. PZ and BH contributed equally. The first draft of the manuscript was written by PZ and BH. QC, XP, ZZ, and WP provide important support for the modification and polishing of the article. Tissue sample acquisition was performed by GT, BH, and PZ. The RNA-seq data results were aided by FY. All authors commented on previous versions of the manuscript and all authors read and approved the final manuscript.

## Funding

This work was supported by the National Undergraduate Innovation Training Program of China (20190034020003 and 20200034020026, BH), the Fundamental Research Funds for the Central Universities of Central South University (2021zzts0383, BH), Hunan Provincial Science and Technology Innovation Plan Project (2020SK53424, XW) and Natural Science Foundation of Hunan Province (2021JJ30957, XW).

## Conflict of Interest

The authors declare that the research was conducted in the absence of any commercial or financial relationships that could be construed as a potential conflict of interest.

## Publisher’s Note

All claims expressed in this article are solely those of the authors and do not necessarily represent those of their affiliated organizations, or those of the publisher, the editors and the reviewers. Any product that may be evaluated in this article, or claim that may be made by its manufacturer, is not guaranteed or endorsed by the publisher.

## Supplementary Material

Supplementary Figure 1 Flow chart of organoid tissue culture and treatment of dosing before immunofluorescence operation.

Supplementary Figure 2 Differences in IL-6 expression between 9 normal lung samples and 9 GGO samples were detected in the validation cohort (Lee H et al.). **P < 0.01

Supplementary Figure 3 The multiple staining immunohistochemical results of CD56 (in yellow) and PD-1(in purple, negative) in normal lung tissue (upper) or GGO lung tissue (lower). Scale bar, 50 µm.

Supplementary Figure 4 High expression of NK cell markers suggests a better prognosis for lung adenocarcinoma (The data are from the TCGA-LUAD database).
