# Supplementary figures and images for: Decreased IL-6 and NK Cells in Early-Stage Lung Adenocarcinoma Presenting as Ground-Glass Opacity
Source: Front Oncol. 2021 Sep 8;11:705888. doi: 10.3389/fonc.2021.705888 (PMC8457009; doi:10.3389/fonc.2021.705888)

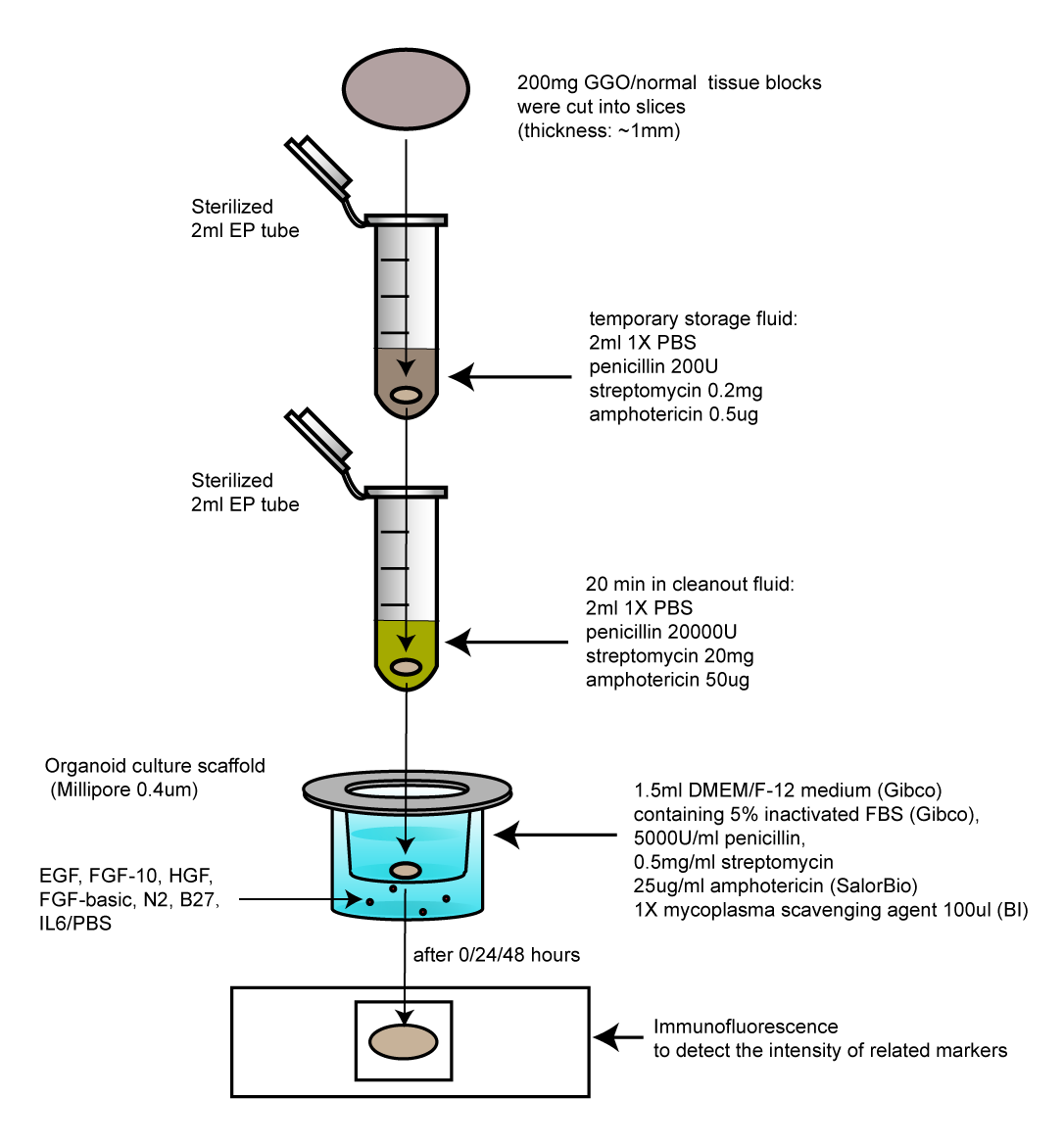

Supplement: Supplementary Figure 1 — Flow chart of organoid tissue culture and treatment of dosing before immunofluorescence operation. [file Image_1.tif]

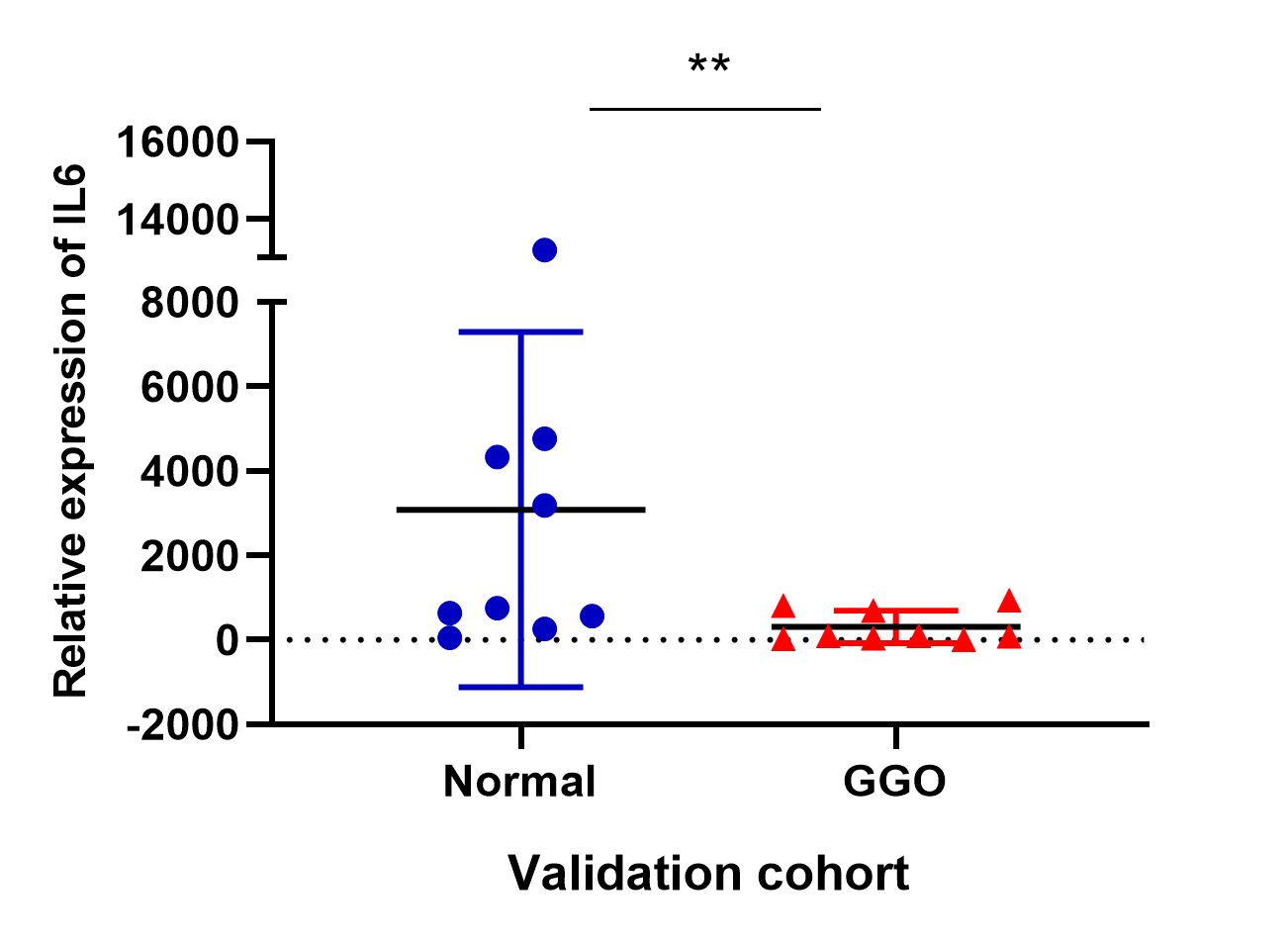

Supplement: Supplementary Figure 2 — Differences in IL-6 expression between 9 normal lung samples and 9 GGO samples were detected in the validation cohort (Lee H et al.). **P < 0.01 [file Image_2.tif]

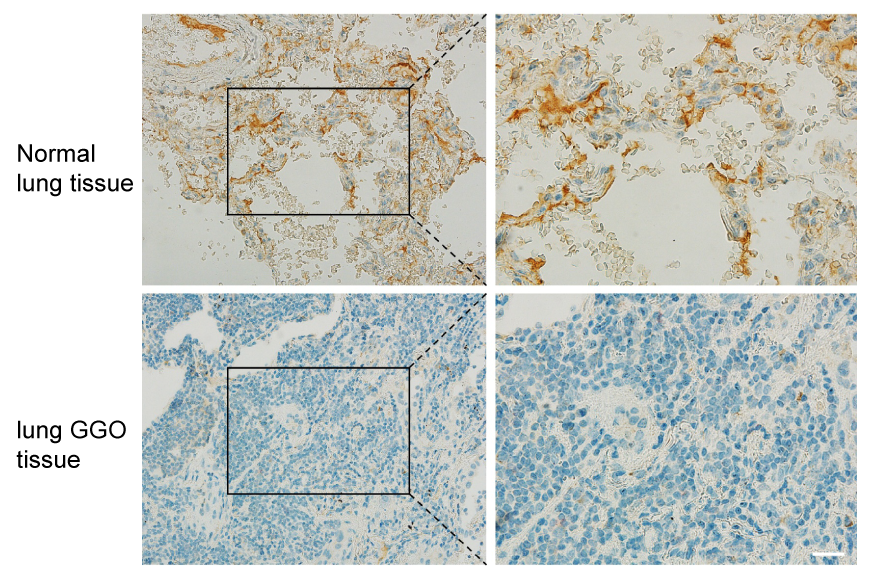

Supplement: Supplementary Figure 3 — The multiple staining immunohistochemical results of CD56 (in yellow) and PD-1(in purple, negative) in normal lung tissue (upper) or GGO lung tissue (lower). Scale bar, 50 µm. [file Image_3.tif]

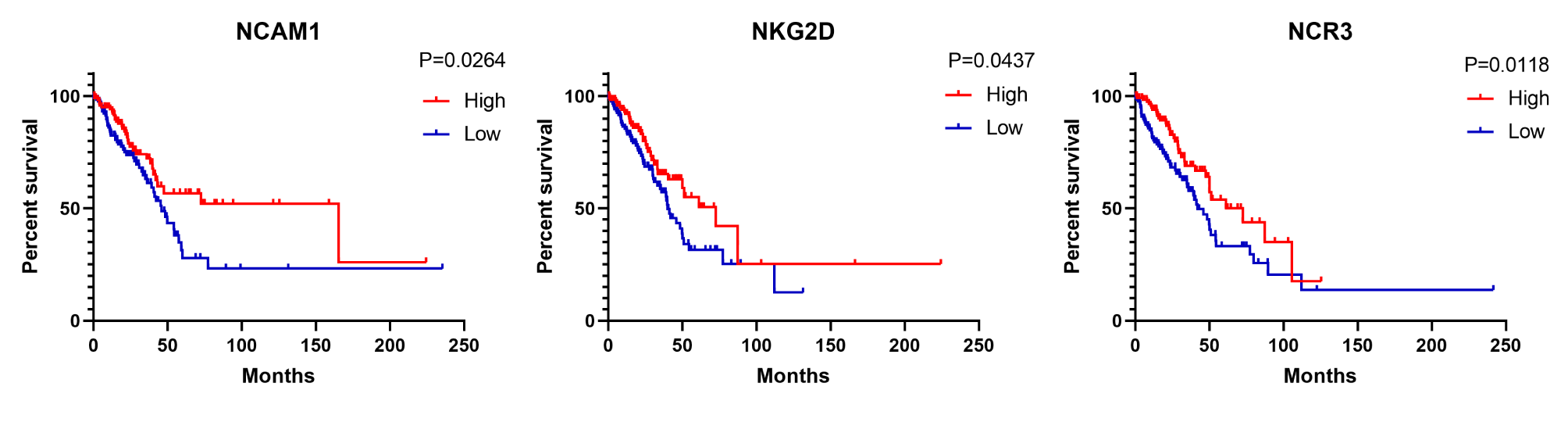

Supplement: Supplementary Figure 4 — High expression of NK cell markers suggests a better prognosis for lung adenocarcinoma (The data are from the TCGA-LUAD database). [file Image_4.tif]
